# Comparison of helminth community of *Apodemus agrarius* and *Apodemus flavicollis* between urban and suburban populations of mice

**DOI:** 10.1007/s00436-017-5609-5

**Published:** 2017-09-14

**Authors:** Dorota Dwużnik, Tomasz Gortat, Jerzy M. Behnke, Alicja Gryczyńska, Małgorzata Bednarska, Antoni S. Mikoszewski, Michał Kozakiewicz, Anna Bajer

**Affiliations:** 1 Department of Parasitology, Faculty of Biology, Institute of Zoology, University of Warsaw, Miecznikowa Street 1, 02-096 Warsaw, Poland; 2 Department of Ecology, Faculty of Biology, Biological and Chemical Research Centre, Institute of Zoology, University of Warsaw, Żwirki i Wigury 101, 02-089 Warsaw, Poland; 3 School of Life Sciences, Faculty of Medicine and Health Sciences, University of Nottingham, Nottingham, UK

**Keywords:** *Apodemus agrarius*, *A. flavicollis*, Helminth community, Urbanization, City park, Forest, Warsaw

## Abstract

The growing human population and the development of urban areas have led to fragmentation and destruction of many natural habitats but have also created new urban habitats. These environmental changes have had a negative impact on many species of plants and animals, including parasite communities. The aim of present study was to compare the helminth communities of *Apodemus flavicollis* and *Apodemus agrarius* in natural and urban habitats. Helminth burdens were assessed in 124 mice, 48 *A. flavicollis*, and 76 *A. agrarius* from two managed forests close to the city boundaries and two city parks within Warsaw, Central Poland. In total, eight species of helminths, Nematoda (*n* = 3), Digenea (*n* = 2), and Cestoda (*n* = 3), were identified. Helminth community structure and prevalence/abundance of individual helminth species differed significantly between the two *Apodemus* species. Overall, prevalence and abundance of helminth species were significantly higher in *A. agrarius* compared to *A. flavicollis*. For *A. flavicollis*, higher prevalence and abundance of helminths were detected in individuals from managed forest habitats in comparison to city parks. In striped field mice, much higher prevalence and mean abundance were recorded in rodents trapped in city parks than in managed forests. This phenomenon may be explained by better adaptation of *A. agrarius*, compared to *A. flavicollis*, to city habitats, resulting in high local densities of mice and the full range of parasite species affecting this host species. Our data confirm also that the established routes of infection exist for selected helminth species in the urban environment.

## Introduction

Urbanization is a social and cultural process reflected in the development of expanding urban areas, an increasing number of cities and a huge increase in the proportion of the total world population living in cities. According to latest estimates, 66% of the human population will live in cities by 2050 (Word Urbanization Prospect 2014). The growing human population and the areas under urbanization have led to fragmentation and destruction of natural habitats but have, in turn, created new artificial urban habitats. Overall, these environmental changes have had a negative impact on many species of plants and animals. Extensive development of cities, public and private transportation and thus the number and surface areas now covered by roads, and pollution of water, soil, and air have all contributed to the extinction of species and loss of biodiversity (Hunter 2007; Luniak 2008; Vitousek et al. 1997).

However, cities do not pose a universal threat to all biodiversity. Urban environments also create many new habitats and ecological niches which can be exploited by species able to adapt to the specific conditions in urban areas (Burger et al. 2004; Etheredge 2013). In contrast to, and parallel with the loss of locally preexisting biodiversity arising from urbanization, recruitment of other wild species of plants and animals to the newly created urban habitats also takes place, a process of adaptation that is referred to as synurbization (Andrzejewski et al. 1978). Adaptation involves habituation to the specific conditions in urban environments, resulting in an ability to reproduce and to establish and maintain population size (Luniak 2004), despite the extremely transformed environment. Transformed environment has an impact not only on food availability, reproduction, population density, and distribution but also on the occurrence of diseases, hence all contributing to the likelihood of survival (Ditchkoff et al. 2006).

Urbanization affects also parasite species, vectored by their hosts into the city system. Adverse factors encountered in the city (especially soil contamination) may have an impact on the parasite community. According to one hypothesis (Lafferty 2012), the reduction in overall biodiversity arising from urbanization is reflected also in a reduction in species richness of parasites. In an urban environment, parasites with complex life cycles are more likely to become locally extinct because they need more than one host to complete their life cycles. Loss of a key intermediate or final host species (vertebrate or invertebrate) will prevent a parasite from existing locally (Lafferty 2012). An example can be found in the work of Lafferty (1993), in which snails *Cerithideopsis californica* residing in an urban pond were not infected with flukes in contrast to those from control pond. The urban pond, adjacent to a car park and highways, was much less frequently visited by waterfowls, the definitive hosts of flukes for which *C. californica* acted as an intermediate host. This resulted in the complete loss of these parasites from the population of snails living in the urban pond (Lafferty 1993).

Alternative hypotheses predict that more parasites should occur in hosts living in more polluted areas (Lafferty 2012). In Moscow, for example, pollution and eutrophication of waters by human activity have been suggested to be the principal cause of higher incidence of flukes in waterfowl and, consequently, a higher number of cases of swimmer’s itch in humans exposed to these waters (Beer and German 1993).

Rodents adapt well to the habitats created by man. The most common species inhabiting even highly urbanized areas such as city centers are brown rats (*Rattus norvegicus*) and house mice (*Mus musculus*/*M. musculus domesticus*). These are regarded as synanthropic species that take advantage of the proximity of human settlements. In many cases, they pose a significant hazard, because they are the source of infection of zoonotic pathogens, including viruses and parasites (Lee et al. 1982; Marangi et al. 2003; Ahmad et al. 2011). With increasing urbanization and expansion of space for the development of cities, other species of rodents have also begun to invade and settle in urban areas. Urban green areas such as parks, squares, suburban wooded areas, or even house gardens all provide suitable habitats that can be exploited by various species of small rodents (Tikhonova et al. 2010).

Striped field mouse *Apodemus agrarius* Pallas, 1771 is a common rodent species throughout Central Europe, including Poland. This species mainly inhabits fields, meadows, edges of forests, roadside scrub parks, and gardens (Kowalski et al. 1984) and is frequently encountered in urban areas of Warsaw where it has been recorded for more than 100 years now (Walecki 1881; Sumiński 1922). *A. agrarius* is known to form stable local populations even in parts of the city subject to the highest anthropopressure (Gortat et al. 2013, 2014, 2015, 2016). Based on research conducted in 1975–1976 in three distinct areas of Warsaw, it has been shown that individuals trapped within the city center possessed the highest mean body weight and were probably the best nourished (Liro 1985). The highest rate of reproduction, especially in autumn, was recorded in a population of this species living in the most urbanized location close to the city center. The number and condition of the rodents that were inspected indicated that *A. agrarius* excels in areas inhabited by humans (Andrzejewski et al. 1978). This species inhabits also many other Polish cities and towns (Stanik and Wołoszyn 2006; Haitlinger 1962; Łopucki et al. 2013).

In marked contrast to *A. agrarius*, the yellow-necked mouse *Apodemus flavicollis* Melchior, 1834 is considered to be a typical forest species of rodent (Kowalski et al. 1984). Although it is a species which prefers forests, it has also been recorded in the Polish capital city. Remnants of yellow-necked mice have been found in tawny owl pellets (*Strix aluco*) collected in Warsaw (Goszczyński et al. 1993). Analyses of *S. aluco* pellets collected in Warsaw in 2003–2006 also indicated the presence of *A. flavicollis* in Warsaw, but mice were not trapped in the city center (Gryz et al. 2008). Occurrence of *A. flavicollis* was recorded for the first time in the northwest part of the capital city in 2008 (Babińska-Werka and Malinowska 2008). Finally, in 2010–2011, *A. flavicollis* was trapped in green areas in the city center of Warsaw (Gortat et al. 2014). It has been also shown that the genetic diversity and flow of genes in populations of *A. flavicollis* and *A. agrarius* decrease from the surrounding countryside in the direction of the city center (Gortat et al. 2015, 2016). Therefore, the degree of urbanization and anthropopressure has a strong influence on the flow of genes in urban populations of both species.

The aim of the present study was to carry out a qualitative and quantitative analysis of the intestinal parasites of the two species, *A. agrarius* and *A. flavicollis*, from urban and suburban sites in Warsaw in order to determine the impact of environment urbanization on their helminth communities.

## Materials and methods

### Study sites

We compared the helminth community of two species of *Apodemus* (*A. agrarius*, *A. flavicollis*) trapped in city parks (urban areas) and managed forests (suburban control areas). Rodents were trapped at four sites: two sites located in Warsaw (site 1, Warszawski Ogród Zoologiczny = Warsaw Zoological Garden = zoo, city park; site 2, Żerań, city park) and at two sites located in the vicinity of Warsaw (site 3, Henryków forest; site 4, Rudka forest). These sites were all situated on the eastern bank of the Vistula River (detailed description in Gortat et al. 2014).

#### Site 1. City park: zoo

Warsaw Zoo was opened in 1928 at Ratuszowa Street in the Praga North-West district and is located on the eastern bank of the Vistula River, just a short distance from the river. Currently, it occupies 32 ha and includes a semi-natural area of the river bank. Adjacent to the zoo, there is another large city park—Park Praski. Both parks are dominated by trees including common aspen (*Populus tremula*), Norway maple (*Acer platanoides*), small leaved lime (*Tilia cordata*), horse chestnut (*Aesculus hippocastanum*), and Norway spruce (*Picea abies*). The zoo can boast an extensive number of species (553 in 2011) and individuals (3566) (http://www.zoo.waw.pl/pl/przewodnik.html) and is very popular with 720,000 visitors in 2011, creating strong anthropopressure in the area occupied by the zoo (www.warszawa.wyborcza.pl/warszawa/1,34889,10903857,Warszawskie_zoo_pobilo_rekord__720_tys__zwiedzajacych.html).

#### Site 2. City park: Żerań

Żerań is the smallest and the newest forest park in Warsaw. It was created after the Second World War when the dunes that it occupies began to be reforested. As a result of the expansion of industrial infrastructure in the Żerań quarter in the last decade, the surface area occupied by the park has decreased by 1.04 ha and currently stands at 16.09 ha. Żerań park is a fragmented forest, surrounded by an industrial complex and limited by roads on its eastern border. In the west, the park borders railway lines, and in the north, it is restricted by the Żerański Canal. In addition, Marywilska Street and a railway line run through the park itself. The forest structure is typical of the plant communities on former farmland that has been abandoned, left to fallow. Common pine (*Pinus sylvestris*) is the dominant tree species, accompanied by silver birch (*Betula pendula*), English oak (*Quercus robur*), black locust (*Robinia pseudoacacia*), and black alder (*Alnus glutinosa*). Being situated just a short distance from industrial plants, large roads, and railway lines, the park is in a heavily polluted region of the city (www.lasymiejskie.waw.pl/Obwod_Bielany-Mlociny).

#### Site 3. Henryków, managed forest

Henryków forest is located in the Białołęka quarter in the northeast part of Warsaw and covers an area of 49.80 ha. Henryków forest is one component of a complex of three forest patches reaching furthest north within the city boundaries of Warsaw. The other two are Dąbrówka forest (27.29 ha), which separates the city from the sewage treatment plant Czajka and Białołęka forest (44.88 ha). Young pine stands dominate in these forests (www.lasymiejskie.waw.pl/Obwod_Bielany-Mlociny).

#### Site 4. Rudka, managed forest

This managed forest near Rudka Sanatoryjna village is a nature reserve created in 1964 for silver fir (*Abies alba*) stand and occupies 125.64 ha. Within the reserve, there are also Norway spruce, European larch (*Larix decidua*), common aspen, and English oak. The undergrowth consists mainly of hazel (*Corylus avellana*), common juniper (*Juniperus communis*), and *Viburnum* spp. (www.mrozy.bip.net.pl/?p=document&action=show&id=1522&bar_id=895).

### Trapping of mice

Trapping sessions took place in September 2010 and 2011 (detailed description in Gortat et al. 2014). Wooden traps were placed along 600-m-long transects with two traps set at 30 trapping points along each transect. Trapping was carried out for seven consecutive days in each location. Captured animals were killed by cervical dislocation, weighed, sexed, and measured. Intestines were stored in 50-ml Falcon tubes in 10% formalin solution. A total of 124 individuals (76 *A. agrarius* and 48 *A. flavicollis*) were used for the parasitological study. Trapping and handling procedures were approved by the First Warsaw Local Ethics Committee for Animal Experimentation (Permission No. 21/2010).

Gastrointestinal tracts were divided into stomach, small intestine, cecum, and large intestine. Each part was cut lengthwise and carefully checked with a stereo microscope at magnifications between × 2.5 and × 10. Nematodes were first placed in 5% glycerol in 70% ethanol for 7 days at a temperature + 37 °C and then embedded in glycerogelatin. Since the gastrointestinal tracts were stored in the 10% formalin solution, in order to rinse the solution off, cestodes and flukes were first placed in water for 24 h. Then, they were flattened and transferred to AFA solution (40% formaldehyde, 95% ethanol, glycerin, glacial acetic acid, distilled water). After 7 days, specimens were washed in 70% EtOH, stained in a 4% solution of borax carmine for 24 h, and differentiated in acidic 70% ethanol. Flatworms were dehydrated using a series of increasing alcohol concentrations (70, < 80, < 85, and < 96% < 2× absolute EtOH), placed in clove oil for 24 h, and embedded in Canada balsam. A Nikon YS-100 microscope was used to study stained specimens at magnifications 40× – 400×.

### Statistical analyses

The statistical approach adopted has been documented comprehensively in our earlier publications (Behnke et al. 2001, 2008a, b; Bajer et al. 2005). For analysis of prevalence (% infected), we used maximum likelihood techniques based on log linear analysis of contingency tables in the software package IBM SPSS Statistics, version 21 (IBM Corporation). HOST (host species at two levels, *A. agrarius* and *A. flavicollis*), SITE (four levels: zoo, Żerań, Henryków, and Rudka), or HABITAT (two levels: city park and managed forest) were used as the factors in models with the presence or absence of helminth considered as a binary factor (0, 1) and referred to as INFECTION. For each level of analysis in turn, beginning with the most complex model, involving all possible main effects and interactions, those combinations that did not contribute significantly to explaining variation in the data were eliminated in a stepwise fashion beginning with the highest level interaction (backward selection procedure). A minimum sufficient model was then obtained, for which the likelihood ratio of chi-square was not significant, indicating that the model was sufficient in explaining the data.

A multifactorial ANOVA was used for the analysis of the mean helminth abundance, using models with normal errors, incorporating HOST (host species at two levels, *A. agrarius* and *A. flavicollis*) and SITE (four levels: zoo, Żerań, Henryków, and Rudka) or HABITAT (two levels: city park and managed forest) as fixed factors.

## Results

### Comparison of helminth community between *A. agrarius* and *A. flavicollis*

Altogether, eight helminth species were recorded in *Apodemus* spp. Total species richness was higher in striped field mice, from which six helminth species (two Cestoda, two Nematoda, and two Digenea) were recovered. In yellow-necked mice, only three species were recorded, two nematodes and one tapeworm (Table 1, *A. flavicollis* and *A. agrarius*). The nematode *Heterakis spumosa* was the only helminth species that occurred in both host species.

**Table 1:** Comparison of the prevalence and abundance of helminths between two host species: *A. flavicollis* and *A. agrarius*

Helminth species	Prevalence (%)	Abundance	
		Mean ± S.E.	Range
*Apodemus flavicollis*			
Nematoda			
*Heterakis spumosa*	30.0	6.17 ± 5.13	0–112
*Heligmosomoides polygyrus*	21.0	1.79 ± 2.02	0–21
Total Nematoda	38.0	7.96 ± 5.66	0–116
Cestoda			
*Microsomacanthus crenata*	3.4	0.27 ± 0.17	0–13
Total Cestoda	3.4	0.27 ± 0.17	0–13
Total helminths	38.0	8.23 ± 2.99	0–116
*Apodemus agrarius*			
Nematoda			
*Heterakis spumosa*	83.0	31.76 ± 4.08	0–271
*Heligmosomoides neopolygyrus*	45.0	8.80 ± 1.60	0–116
Total Nematoda	83.0	40.57 ± 4.50	0–271
Cestoda			
Adult Cestoda	2.6	0.26 ± 0.14	0–56
*Rodentolepis fraterna*	2.6	0.26 ± 0.14	0–56
Larval Cestoda			
*Mesocestoides* sp.	1.3	4.03 ± 3.16	0–306
Total Cestoda	3.9	4.78 ± 4.80	0–306
Digenea			
*Plagiorchis* sp.	16.0	0.84 ± 0.39	0–37
*Branchylaima* sp.	1.3	0.01 ± 0.01	0–1
Total Digenea	17.0	0.86 ± 0.50	0–37
Total helminths	83.0	46.2 ± 5.56	0–351

Accordingly, mean species richness (MSR) was almost three times higher in *A. agrarius* in comparison to *A. flavicollis* (0.521 ± 0.13 for *A. flavicollis* and 1.487 ± 0.10 for *A. agrarius*), and this difference was significant (main effect of HOST on MSR: *F*_1, 123_ = 34.54, *P* < 0.001).

The overall prevalence of helminths was twice as high in *A. agrarius* compared with *A. flavicollis* (83 vs. 38%) (HOST × INFECTION: *χ* ^2^_1_ = 27.01, *P* < 0.001), and the mean helminth abundance was over five times higher in striped field mice (Table 1, *A. flavicollis* and *A. agrarius*) (main effect of HOST on helminth abundance: *F*_1, 123_ = 17.41, *P* < 0.001).

As nematodes were the most common and numerous helminths in both *A. agrarius* and *A. flavicollis*, similar differences were seen in overall nematode prevalence and abundance between the two host species (Table 1, *A. flavicollis* and *A. agrarius*) (HOST × INFECTION: *χ* ^2^ = 27.01, *df* = 1, *P* < 0.001; main effect of HOST on nematode abundance: *F*_1, 123_ = 20.39, *P* < 0.001).

In the case of cestodes, three different species were recorded: one in *A. flavicollis* and two (one adult and one larval form) in *A. agrarius*, but the overall prevalence of cestodes was less than 5%, and differences in prevalence and abundance between host species were not significant (Table 1, *A. flavicollis* and *A. agrarius*).

Two species of Digenea were found and only in *A. agrarius* (Table 1, *A. flavicollis* and *A. agrarius*).

The prevalence of several individual parasite species differed significantly between the two host species. A significant difference in the prevalence of *Heligmosomoides* spp. was found to be more than twice as high in *A. agrarius* compared with *A. flavicollis* (HOST × INFECTION: *χ* ^2^_1_ = 7.66, *P* = 0.006), although it has to be emphasized here that each of these *Apodemus* spp. carried its own and different species of *Heligmosomoides* (*Heligmosomoides polygyrus* in *A. flavicollis* and *Heligmosomoides neopolygyrus* in *A. agrarius*; Zaleśny et al. 2014). Abundance was also higher in *A. agrarius* (four times) in comparison to *A. flavicollis* (main effect of HOST on abundance of *Heligmosomoides*: *F*_1, 123_ = 7.41, *P* = 0.007) (Table 1, *A. flavicollis* and *A. agrarius*). Similar differences between the two hosts were seen for the prevalence and abundance of *H. spumosa* (Table 1, *A. flavicollis* and *A. agrarius*) (HOST × INFECTON: *χ* ^2^_1_ = 37.07, *P* < 0.001; main effect of HOST on abundance of *H. spumosa*: *F*_1, 123_ = 15.24, *P* < 0.001). In the case of *H. spumosa*, prevalence was almost three times higher and abundance was up to five times higher in striped field mice in comparison to yellow-necked mice (Table 1, *A. flavicollis* and *A. agrarius*).

Because of these highly significant differences between the hosts, and our finding that they only shared one species of helminth, in subsequent analyses, data for each host species were analyzed separately.

### Comparison of helminth communities of *A. flavicollis* and *A. agrarius* between city parks and managed forests

#### *A. flavicollis*

Total species richness was three for 13 individuals of *A. flavicollis* trapped in city parks. Only two species of helminths (*H. polygyrus* and *H. spumosa*) were recorded in 35 individuals of *A. flavicollis* originating from the managed forests.

Mean species richness was slightly lower in mice inhabiting city parks in comparison to mice from the forests (0.46 ± 0.22 vs. 0.54 ± 0.13); however, this difference was not significant (NS).

Although the prevalence of helminths was about 10% lower in mice from the city parks in comparison to mice from the managed forests, the difference was NS (Table 2). Similarly, lower abundance of helminths was detected in mice from city parks in comparison to mice from forests, but the difference was not significant (main effect of HABITAT on abundance of helminths: *F*_1, 47_ = 1.51, *P* = 0.225) (Table 2).

**Table 2:** Comparison of the prevalence and abundance of helminths between two habitats for yellow-necked mouse

Helminth species	City parks, *n* = 13			Managed forests, *n* = 35		
	Prevalence (%)	Abundance		Prevalence (%)	Abundance	
		Mean ± S.E.	Range		Mean ± S.E.	Range
Nematoda						
*H. spumosa*	30.8	1.08 ± 5.08	0–6	28.6	8.06 ± 3.10	0–112
*H. polygyrus*	7.7	0.15 ± 1.29	0–2	25.7	2.40 ± 0.79	0–21
Total Nematoda	30.8	1.23 ± 5.67	0–8	40.0	10.46 ± 3.46	0–116
Cestoda						
*M. crenata*	7.7	1.0 ± 0.32	1	0	0	0
Total Cestoda	7.7	1.0 ± 0.32	1	0	0	0
Total helminths	30.8	2.23 ± 5.72	0–21	40.0	10.46 ± 3.48	0–116

The prevalence of nematodes was again about 10% lower in mice from the city parks in comparison to mice from the managed forests, but the difference was NS (Table 2). Similarly, the abundance of nematodes was lower in mice from city parks in comparison to mice from forests (eight times higher in forest mice), but this difference was also not significant (main effect of HABITAT on abundance of nematodes: *F*_1, 47_ = 1.93, *P* = 0.171) (Table 2).

One species of Cestoda (*Microsomacanthus crenata*) was found in one mouse *A. flavicollis* trapped in the zoo city park. No tapeworms were recorded in mice from the forests (Table 2).

Similar patterns were observed among individual nematode species (Table 2). Although the prevalence of *H. spumosa* was almost identical in mice from both city parks and managed forests, mean abundance was numerically higher in mice trapped in the latter (Table 2) although this was not statistically significant (main effect of HABITAT on abundance of *H. spumosa*: *F*_1, 47_ = 1.36, *P* = 0.247).

Similar tendencies were observed for the prevalence and abundance of *H. polygyrus*, with both parameters numerically higher in mice from the forests (HABITAT × INFECTION: *χ* ^2^_1_ = 2.17, *P* = 0.140; main effect of HABITAT on abundance of *H. polygyrus*: *F*_1, 47_ = 2.21, *P* = 0.144) (Table 2).

#### *A. agrarius*

Total species richness was six for both the 36 individuals of *A. agrarius* from city parks and also for the 40 individuals from managed forest, with four common species and two species occurring only in one type of habitat: larval *Mesocestoides* sp. were recovered only from one mouse from one of the managed forest sites, and a single individual of *Branchylaima* sp. was found in a mouse from one of the city parks (Table 3).

**Table 3:** Comparison of the prevalence and abundance of helminths between two habitats for stripped field mouse

Helminth species	City parks, *n* = 36			Managed forests, *n* = 40		
	Prevalence (%)	Abundance		Prevalence (%)	Abundance	
		Mean ± S.E.	Range		Mean ± S.E.	Range
Nematoda						
*H. spumosa*	91.7	32.78 ± 7.21	0–150	75.0	30.85 ± 6.84	0–271
*H. neopolygyrus*	61.1	14.53 ± 2.76	0–116	30.0	3.65 ± 2.63	0–31
Total Nematoda	91.7	47.31 ± 7.87	0–150	75.0	34.50 ± 7.45	0–271
Cestoda						
Adult Cestoda						
*R. fraterna*	2.8	0.28 ± 1.07	0–1	2.5	1.40 ± 1.02	0–56
Larval Cestoda						
*Mesocestoides* sp.	0	0	0	2.5	7.65 ± 5.55	0–306
Total Cestoda	2.8	0.03 ± 5.92	0–1	5.0	9.05 ± 5.62	0–306
Digenea						
*Plagiorchis* sp.	27.8	1.69 ± 0.72	0–2	5.0	0.08 ± 0.68	0–37
*Branchylaima* sp.	2.8	0.28 ± 0.19	0–1	0	0	0
Total Digenea	30.6	1.72 ± 0.72	0–2	5.0	0.08 ± 0.68	0–37
Total helminths	91.7	49.06 ± 10.19	0–165	75.0	43.63 ± 9.66	0–351

Mean species richness was significantly higher in mice inhabiting city parks in comparison to mice from the managed forests (1.86 ± 0.15 vs. 1.15 ± 0.14, respectively) (main effect of HABITAT on MSR: *F*_1, 75_ = 11.96, *P* = 0.001).

The prevalence of helminths was higher in mice from the city parks (92%) than in individuals trapped in the managed forests (75%) (HABITAT × INFECTION *χ* ^2^_1_ = 3.91, *P* = 0.048) (Table 3). However, the mean abundance of helminths was very similar in both types of habitats (Table 3).

As nematodes were the most common and numerous helminth taxon in both types of habitats, similar differences were seen in the overall nematode prevalence and abundance between city parks and managed forests (Table 3) (HABITAT × INFECTION: *χ* ^2^_1_ = 3.91, *P* = 0.048), with prevalence higher in mice caught in city parks. Numerically, the mean abundance was also higher in mice from the city parks but this was not significant (*F*_1, 75_ = 1.40, *P* = 0.241) (Table 3).

The prevalence of cestodes was very low in both types of habitat (< 5%), so the differences in prevalence/abundance between habitats were not significant (Table 3). However, the prevalence of flukes was higher in mice from city parks in comparison to mice from the managed forests (HABITAT × INFECTION: *χ* ^2^_1_ = 9.35, *P* = 0.002) (Table 3). Similarly, abundance was numerically higher in rodents from city parks compared to rodents from the managed forests, but this was not statistically significant (main effect of HABITAT on abundance of flukes: *F*_1, 75_ = 2.79, *P* = 0.10) (Table 3).

Similar trends were observed in the prevalence and abundance of the three most common individual parasite species (*H. neopolygyrus*, *H. spumosa*, and *Plagiorchis* sp.). The prevalence of *H. neopolygyrus* was twice as high in mice from the city parks in comparison to mice from the managed forests (Table 3) (HABITAT × INFECTION: *χ* ^2^_1_ = 7.53, *P* = 0.006). The abundance of *H. neopolygyrus* was three times higher in mice from the city parks compared to the forests (main effect of HABITAT on abundance of *H. neopolygyrus*: *F*_1, 75_ = 8.09, *P* = 0.006) (Table 3).

Although significantly higher prevalence of *H. spumosa* was observed in mice from the city parks (HABITAT × INFECTION: *χ* ^2^_1_ = 3.91, *P* = 0.048), the abundance of *H. spumosa* was very similar in mice from both types of habitats (NS) (Table 3).

A similar pattern was also noted for *Plagiorchis* sp.: prevalence was several times higher in mice from the city parks (HABITAT × INFECTION: *χ* ^2^_1_ = 7.86, *P* = 0.005), but the difference in abundance was NS (Table 3).

### Comparison of helminth communities of *A. flavicollis* and *A. agrarius* between four sites

Each type of habitat was represented by two sites. To control for the individual differences between the sites, this analysis was conducted for each host species separately.

### *A. flavicollis*

Of the total species richness in two city parks, only one species of helminths (*H. spumosa*) was recorded among 12 yellow-necked mice trapped at the most urbanized site in Żerań. However, single individual trapped in the zoo harbored three species of helminthes (*H. polygyrus*, *H. spumosa*, and *M. crenata*). In mice at both forest sites (Henryków, *n* = 18; Rudka, *n* = 17), two species of helminths were recorded, *H. polygyrus* and *H. spumosa*.

**Fig. 1:**
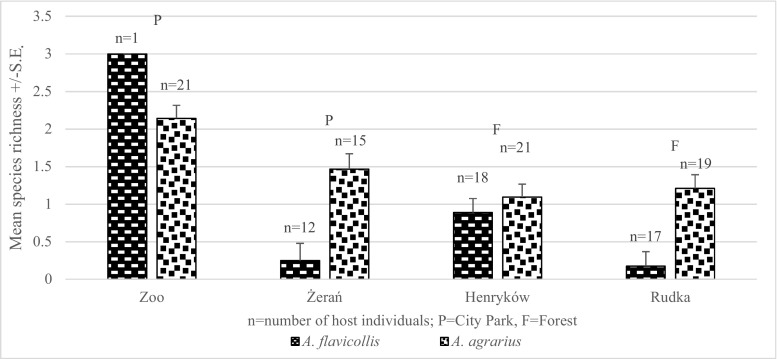
Mean species richness by site for *A. flavicollis* and *A. agrarius*

The highest MSR (0.889 ± 0.144) was observed in yellow-necked mice from the Henryków forest. However, similar low MSR was obtained for mice from Żerań (city park, 0.250 ± 0.18) and from Rudka (managed forest, 0.176 ± 0.15) (*F*_1, 47_ = 10.20, *P* < 0.001) (Fig. 1). The mean species richness could not be calculated for the zoo park as only one mouse was trapped there. Because of that, the zoo site was not involved in the analyses of site effect on prevalence and abundance (no possibility to calculate these parameters for one rodent).

**Fig. 2:**
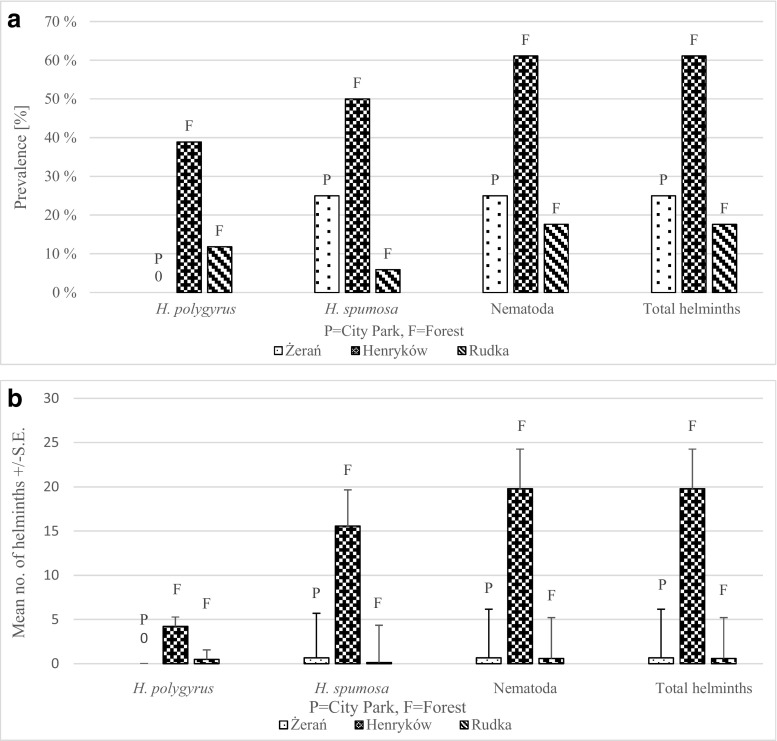
Comparison of prevalence (**a**) and abundance (**b**) of parasites in *A. flavicollis* between three sites

There were significant differences in the prevalence of helminths between three sites (SITE × INFECTION: *χ* ^2^_2_ = 8.12, *P* = 0.017). The prevalence of helminths was the highest in Henryków forest, and much lower but comparable prevalence of helminths was recorded in Rudka forest and Żerań city park (Fig. 2a). Similar significant differences were found in abundance of helminths between three sites (main effect of SITE on abundance of helminths: *F*_2, 47_ = 3.94, *P* = 0.02) The highest abundance of helminths was observed in Henryków forest, and again, almost identical abundance was recorded in Żerań city park and in Rudka forest (Fig. 2b).

Because of the high input of nematodes to helminth burden, the output of statistical analysis was very similar for INFECTION of nematodes and helminths. The prevalence of nematodes was the highest in Henryków forest and lower and similar in Rudka forest site and in Żerań city park (SITE × INFECTION: *χ* ^2^_2_ = 8.12, *P* = 0.017) (Fig. 2a). The highest abundance of nematodes was observed in Henryków forest, and identical abundance was recorded in Żerań city park and in Rudka forest (main effect of SITE on abundance of nematodes: *F*_2, 47_ = 5.64, *P* = 0.007) (Fig. 2b).

There were also significant differences in the prevalence and abundance of *H. polygyrus* between three sites (SITE × INFECTION: *χ* ^2^ = 8.12, *P* = 0.017; main effect of SITE on abundance of *H. polygyrus*: *F*_2, 47_ = 4.42, *P* = 0.018). At the most urbanized site in Żerań, no mice infected with *H. polygyrus* were found (0%); the prevalence of *H. polygyrus* reached almost 40% in Henryków and 20% in Rudka forest (Fig. 2a). One mouse from the zoo was infected with this species. A similar pattern was observed for the abundance of *H. polygyrus* (Fig. 2b).

There were also significant differences in the prevalence and abundance of *H. spumosa* between three sites (SITE × INFECTION: *χ* ^2^_2_ = 9.38, *P* = 0.009; site on abundance of *H. spumosa*: *F*_2, 47_ = 4.24, *P* = 0.021). The prevalence of *H. spumosa* was the highest in Henryków forest, half lower in Żerań park, and the lowest (< 10%) in Rudka forest (Fig. 2a). One mouse from the zoo was infected with this species. A similar pattern was observed for the abundance of *H. spumosa* (Fig. 2b).

### *A. agrarius*

For this host species, the differences between four study sites were analyzed (Fig. 1). The highest number of helminth species (total species richness = 5) was recorded for the group of 21 field mice trapped in the Warsaw Zoo (*H. neopolygyrus*, *H. spumosa*, *Plagiorchis* sp., *Rodentolepis fraterna*, *Branchylaima* sp.). Four species of helminths (*H. neopolygyrus*, *H. spumosa*, *Mesocestoides* sp., and *Plagiorchis* sp.) were found in Henryków forest. Three species of helminths (*H. neopolygyrus*, *H. spumosa*, and *Plagiorchis* sp.) were found in 15 mice in the most urbanized site in Żerań park, and three species of helminths (*H. neopolygyrus*, *H. spumosa*, and *R. fraterna*) in 19 mice in Rudka forest were found.

The highest MSR was observed in field mice from the city parks: the Warsaw Zoo and Żerań (2.143 ± 0.19 and 1.467 ± 0.23, respectively) (Fig. 1). The lowest MSR was obtained for mice at Henryków and Rudka forest sites (main effect of SITE on MRS: *F*_3, 75_ = 5.96, *P* = 0.001) (Fig. 1).

**Fig. 3:**
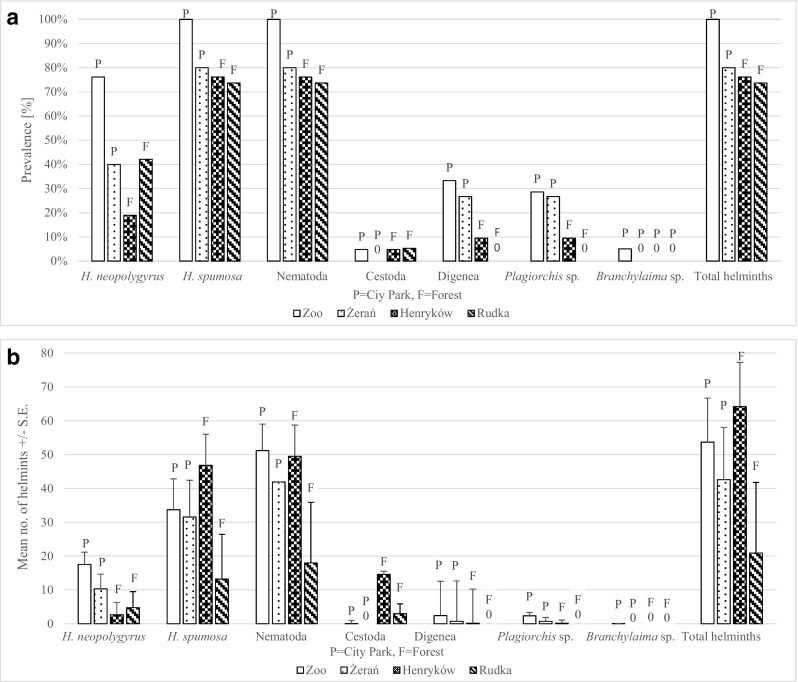
Comparison of prevalence (**a**) and abundance (**b**) of parasites in *A. agrarius* between four sites

There were significant differences in the prevalence of helminths between four sites (SITE × INFECTION: *χ* ^2^_3_ = 9.58, *P* = 0.022). The prevalence of helminths was the highest in mice from the zoo (100%) and the lowest in Henryków and Rudka forests (Fig. 3a). No significant differences in abundance of helminths were observed between four sites although again abundance was the lowest in Rudka (Fig. 3b).

Again, because of the high contribution of nematodes to helminth burden, the output of statistical analysis was very similar for the prevalence/abundance of nematodes and helminths. The prevalence of nematodes was the highest in the zoo (100%) and the lowest in Rudka forest (SITE × INFECTION: *χ* ^2^_3_ = 9.58, *P* = 0.022) (Fig. 3a). The abundance of nematodes tended to be the highest in the zoo and the lowest in Rudka (main effect of SITE on abundance of nematodes: *F*_3, 75_ = 2.15, *P* = 0.10) (Fig. 3b).

There were significant differences in the prevalence of Digenea between four sites (SITE × INFECTION: *χ* ^2^_3_ = 12.21, *P* = 0.007). The prevalence of flukes was the highest in both city parks, the zoo, and Żerań. Much lower prevalence was recorded in Henryków forest, and no flukes were found in mice in Rudka (Fig. 3a). No significant differences in abundance of Digenea were observed between four sites (Fig. 3b). Because of the high contribution of *Plagiorchis* sp. to Digenea burden, the output of statistical analysis and pattern of differences were very similar for the prevalence/abundance of *Plagiorchis* sp. and Digenea (Fig. 3ab). Only differences in prevalence between four sites were significant (SITE × INFECTION: *χ* ^2^_3_ = 9.58, *P* = 0.022).

Although *H. neopolygyrus* occurred at all studied sites, there were significant differences in prevalence between four sites (SITE × INFECTION: *χ* ^2^_3_ = 14.96, *P* = 0.002) (Fig. 3a). The highest prevalence was observed in mice from Warsaw Zoo and the lowest in Henryków forest. There were also significant differences in abundance of *H. neopolygyrus* between four sites (main effect of SITE on abundance of *H. neopolygyrus*: *F*_3, 75_ = 3.28, *P* = 0.026). In two city parks, in the zoo and Żerań, the abundance of *H. neopolygyrus* was higher than that in Henryków and Rudka (Fig. 3b).

There were significant differences in the prevalence of *H. spumosa* between four sites (SITE × INFECTION: *χ* ^2^_3_ = 9.58, *P* = 0.022). The prevalence of *H. spumosa* was the highest in the zoo (100%) and Żerań and lower in Henryków and Rudka forests (Fig. 3a). No significant differences in the abundance of *H. spumosa* were observed between four sites although the abundance was the lowest at Rudka forest site (NS) (Fig. 3b).

## Discussion

The main finding of our study is the contrasting pattern of the impact of urbanization on helminth communities in *A. agrarius* and *A. flavicollis*. We have confirmed also that the helminth community structure and prevalence/abundance of individual helminth species differ significantly between the two *Apodemus* species. The prevalence and abundance of total helminth species were significantly higher in *A. agrarius* compared to those in *A. flavicollis*, a finding that is consistent with data obtained by earlier researchers (Mazeika 2003; Kucia et al. 2006; Ondrikova et al. 2010; Biejlić-Čabrilo et al. 2013).

Analyses of total and mean species richness, prevalence, and abundance of higher helminth taxa and individual helminth species indicated clearly that the helminth community of *A. flavicollis* in city parks is deprived (less numerous and diverse) in comparison to mice from a non-urban environment as represented by the managed forest sites in our study. However, the species richness was higher in the city. This finding is in agreement with earlier work on a range of other species and emphasizes the negative effect on species richness of human-derived changes in habitat structure (Hunter 2007; Czech et al. 2000). Generally, higher species richness of helminths has been observed in hosts colonizing natural non-urbanized environments (Chace and Walsh 2006; Sitko and Zaleśny 2014; Calegaro-Marquez and Amato 2014).

However, for the second *Apodemus* species, the pattern was clearly reversed; striped field mice from the city parks were characterized by higher prevalence and abundance of helminths such as *H. spumosa* and *H. neopolygyrus*, in comparison to mice trapped outside the Warsaw area. There may be two possible explanations for this finding. Firstly, it may be that *A. agrarius* suffer from environment pollution that makes them more vulnerable for helminth invasion. However, it seems rather that *A. agrarius* are very well adapted to city habitats. After all, *A. agrarius* was recognized as a common member of the urban mammalian fauna many years ago (Walecki 1881; Sumiński 1922), and a more recent study (Liro 1985) has revealed that urban striped field mice are generally in good condition and achieve high population densities in urban areas. Efficient transmission and long-term maintenance of their characteristic helminth parasites is therefore not unexpected.

In contrast, *A. flavicollis*, typically a forest-dwelling rodent, is a relatively new colonizer of urban areas in which it is still regarded as a rare species (Wypiórkiewicz 2005). This rodent was detected in Warsaw for the first time approximately 30 years ago (Andrzejewski et al. 1978; Gliwicz 1980; Babińska-Werka and Malinowska 2008) and currently inhabits several green patches in this city, but its numbers are still low (Gortat et al. 2014). The population density of *A. flavicollis* at our trapping sites in Warsaw was markedly lower than that of *A. agrarius* (Gortat et al. 2014), and therefore, fewer individuals were successfully trapped for the present study. Such low-density host populations are not likely to support efficient transmission of helminths with direct life cycles, and hence, the prevalence/abundance of relevant species is predictably lower than that among individuals living in their natural environment in forests in the countryside. The relationship between host population density and prevalence and abundance of parasitic nematodes is well established (Arneberg et al. 1998).

Interestingly, in both mouse species, a dominant member of the helminth community was *H. spumosa*, a parasite commonly found in *Rattus* sp. (Firlotte 1948; Gomez Villafañe et al. 2008; Milazzo et al. 2010). This nematode has also been recovered from both *A. agrarius* and *A. flavicollis* and from other rodents in urban sites in the vicinity of Wrocław, in SW Poland (Hildebrand et al. 2004, 2009; Zaleśny et al. 2010). Nematode *H. spumosa* is regarded as a cosmopolitan parasite of commensal rodents, mainly rats but also house mice, which constitute the main reservoir for this parasite and contaminate the urban environment with eggs to which, inevitably, other colonizing rodents are exposed when they enter the urban environment. In contrast, in our long-term studies in rural woodland sites in the Mazury lake district, this species has never been recovered from *A. flavicollis* or *Apodemus sylvaticus*. In this sylvatic environment, the oxyuroid nematode *Syphacia* spp. were the dominant intestinal nematodes (Bajer 2002; Kuliś-Małkowska 2006). In Żerań city park, a site that is subject to the highest anthropopressure, *H. spumosa* was the only species of parasite recovered from *A. flavicollis*. Because of the sympatric occurrence of the reservoir hosts (*Mus* and *Rattus* spp.) with the invasive *Apodemus* spp. in Warsaw, *H. spumosa* may have adapted to the latter hosts, but the extent to which *H. spumosa* from these different hosts differ and whether they constitute cryptic subspecies can only be resolved by genetic studies and comparison of key informative genetic sequences isolated from worms from these hosts. Analyses of 18S rRNA gene fragment of *H. spumosa* originating from three rodent hosts (*A. agrarius*, *A. flavicollis*, and *R. norvegicus*) from Wrocław area revealed no differences in these sequences, supporting hypothesis of spillover of *H. spumosa* from a commensal rodent to *Apodemus* spp. (Zaleśny et al. 2010). Although our study sites were located in Central Poland, about 300 km apart from Wrocław area in SW Poland, the differences in infection pattern of *H. spumosa* between the two *Apodemus* species were very similar. Interestingly, in the latter study as in our study, the prevalence of *H. spumosa* was 3× higher and abundance was even up to 20× higher in field mice in comparison to yellow-necked mice (Zaleśny et al. 2010); thus, the authors concluded that *A. agrarius* is typical and *A. flavicollis* is only a new auxiliary host of this species.

In addition, to host density-dependent transmission of helminths as an explanation for the difference in parasite burdens in *A. flavicollis* and *A. agrarius* in city parks, it is also possible that the polluted urban environment in Warsaw affected immunocompetence of these rodents, resulting in a greater susceptibility to infective agents, including intestinal helminths. Pollution, noise, and stress can all weaken the immune system and promote infection by parasites (Jancova et al. 2006). Other studies have shown that high concentrations of heavy metals such as iron, zinc, and cadmium can be detected in internal organs of *A. flavicollis* caught in very contaminated environments, and the levels recorded are sufficient to impair the normal functioning of the immune system (Jancova et al. 2006). In our study, Warsaw Zoo located in the East Prague District of the city is a prime example of a highly polluted site subject to air and traffic pollution, dust, and considerable noise (Podawca and Rutkowska 2013). Likewise, Żerań city park is classified as one of the most polluted parks in Warsaw (www.lasymiejskie.waw.pl/Obwod_Bielany-Mlociny). Nevertheless, it is also worth bearing in mind that some of the helminth species that we identified appear to be able to cope well in polluted urban environments (e.g., some Digenea; Beer and German 1993), and it is pertinent that a surprisingly high prevalence of Digenea was noted in *A. agrarius*, in our study, perhaps also reflecting good adaptation of these flukes to polluted environments (Beer and German 1993; Lafferty and Kuris 1999).

Differences in helminth communities between the four study sites for two *Apodemus* species are also interesting to consider. The Rudka forest site was characterized by a relatively low species richness and prevalence/abundance of helminths, especially in comparison to Henryków forest. Rudka is a component of an extensive forest complex so we expected the highest species richness for *A. agrarius* and *A. flavicollis* at this site. However, in relatively undisturbed natural habitats, rodents are preyed upon by a large range of natural enemies, including carnivorous mammals and birds such as raptors and owls, which may eliminate weaker, highly parasitized individuals from the populations. It is also interesting and perhaps remarkable and unexpected that Warsaw Zoo constituted an excellent environment for the transmission of helminths in *A. agrarius* (the highest prevalence and abundance).

In conclusion, the prevalence and mean abundance of helminths in *A. agrarius* are higher in city parks than in the managed forests. In *A. flavicollis*, a species regarded as only relatively recently invading cities, helminth communities were considerably poorer in urban individuals compared to rodents from the managed forests. However, the species richness was higher in the city park. Further, long-term research on the ecology of city invasion by *A. flavicollis* is required to determine whether this species will become eventually as synurbic as *A. agrarius*, and how this will affect the intestinal parasites’ community typically harbored by this host.
